# Electrodeposited Polyaniline Nanofibers and MoO_3_ Nanobelts for High-Performance Asymmetric Supercapacitor with Redox Active Electrolyte

**DOI:** 10.3390/polym12102303

**Published:** 2020-10-08

**Authors:** Wei Meng, Yanlin Xia, Chuanguo Ma, Xusheng Du

**Affiliations:** 1 Institute of Advanced Wear & Corrosion Resistance and Functional Materials, Jinan University, Guangzhou 510632, China; 15385782397@163.com (W.M.); yanlin_xia@163.com (Y.X.); 2 Guangxi Key Laboratory of Information Materials, Guilin University of Electronic Technology, Guilin 541004, China; machuanguo@guet.edu.cn

**Keywords:** polyaniline nanofiber, electro-deposition, MoO_3_ nanobelt, redox active electrolyte, asymmetric supercapacitor

## Abstract

Transition molybdenum oxides (MoO_3_) and conductive polymer (polyaniline, PANI) nanomaterials were fabricated and asymmetric supercapacitor (ASC) was assembled with MoO_3_ nanobelts as negative electrode and PANI nanofibers as a positive electrode. Branched PANI nanofibers with a diameter of 100 nm were electrodeposited on Ti mesh substrate and MoO_3_ nanobelts with width of 30–700 nm were obtained by the hydrothermal reaction method in an autoclave. Redox active electrolyte containing 0.1 M Fe^2+/3+^ redox couple was adopted in order to enhance the electrochemical performance of the electrode nano-materials. As a result, the PANI electrode shows a great capacitance of 3330 F g^−1^ at 1 A g^−1^ in 0.1 M Fe^2+/3+^/0.5 M H_2_SO_4_ electrolyte. The as-assembled ASC achieved a great energy density of 54 Wh kg^−1^ at power density of 900 W kg^−1^. In addition, it displayed significant cycle stability and its capacitance even increased to 109% of the original value after 1000 charge–discharge cycles. The superior performance of the capacitors indicates their promising application as energy storage devices.

## 1. Introduction

As an important type of energy storage device, the super-capacitor has been widely used in many electronic devices, including the automobile braking energy recovery/release system, due to its superior power density, fast charging speed, and a wide range of working temperature. However, the relatively low energy density and poor long-time discharging performance restricted its application [1]. Two approaches were usually utilized in order to increase the energy density of the capacitor: one is enhancing its electric capacitance directly; another is enlarging its operation voltage, as the energy density of the capacitor is proportional to the square of the operation voltage theoretically. Asymmetric supercapacitor could take advantage of two different electrodes to extend the working potential window of the device. Notably, the operating voltage of the full device could be maximized even beyond the thermodynamic decomposition voltage of aqueous electrolyte in certain cases [2]. Therefore, an asymmetric super-capacitor was developed and assembled by matching different anode and cathode materials in order to realize a wider working voltage range [3,4,5]. Moreover, transition metal oxides or conductive polymers that theoretically possessed remarkable pseudo-capacitance were usually studied as electrode materials in the capacitors [6,7]. The electrochemical capacitive performance of the transition metal oxides, such as manganese dioxide (MnO_2_), ruthenium oxide (RuO_2)_, cobalt oxide (Co_3_O_4_), and molybdenum oxide (MoO_3_), have been extensively investigated [8,9,10,11]. In the meanwhile, polyaniline (PANI) is one of the most studied conductive polymers due to its simple synthetic method, low-cost, and high pseudo-capacitance attributed to various redox state [12]. However, its volume change originated from the expansion/shrinkage during the charge/discharge process can lead to the deformation of electrode structure and poor cycle stability that hindered its application in supercapacitors. 

An innovative method was recently developed through affording the electrolyte with high electrochemical activity in order to further improve the electrochemical performance of the capacitors [13,14,15,16]. Plenty of excellent works regarding the electrochemically active electrolyte have been reported. Ren et al. reported that the specific capacitance of PANI in its symmetric capacitor was promoted to be 1062 F g^−1^ at 2 A g^−1^ by utilizing the redox active electrolyte of 1 M H_2_SO_4_ + 0.8 M Fe^3+^/Fe^2+^ [17]. By doping PANI electrode materials with iron ions, an even higher specific capacitance of this conductive polymer was achieved in the electrochemically active electrolyte in previous work [18]. Senthilkumar et al. reported a nearly two-fold improved specific capacitance of 912 F g^−1^ and energy density of 19.04 Wh kg^−1^ by adding 0.08 M KI to 1 M H_2_SO_4_ electrolyte [16]. The high area specific capacitance of CNFs/Fe_2_O_3_/SS mesh electrode in the redox active electrolyte was also demonstrated in our recent work [4]. Although the transition metal oxides and conductive polymers are well-known electrode materials with high theoretical specific capacitance, little information regarding the utilization of them together to assemble asymmetric super-capacitor (ASC) with electrochemical redox active electrolyte is available so far.

Integration on the redox active electrolyte and asymmetric super-capacitor could be an efficient method for promoting the energy density of SCs without sacrificing the power density and cycling stability [2,14]. Nilesh R. Chodankar et al. studied the electrochemical properties of the MWCNTs/MnO_2_ electrode in the redox-active electrolyte (K_3_[Fe(CN)]_6_ modified aqueous Na_2_SO_4_ electrolyte) and achieved a notable specific capacitance of 1012 F g^−1^ at 2 mA cm^−1^, which is 1.5 fold larger than that measured in the conventional Na_2_SO_4_ electrolyte. Moreover, for the MWCNTs/MnO_2_//Fe_2_O_3_ asymmetric capacitor equipped with this hybrid electrolyte, an excellent performance with maximum specific capacitance of 226 F g^−1^ and energy density of 54.39 Wh kg^−1^ at power density of 667 W kg^−1^ was obtained [19].

In this article, transition metal oxide (MoO_3_) and conductive polymer (PANI) nano-materials were both fabricated and utilized as negative and positive electrodes in an ASC with redox active electrolyte, respectively. MoO_3_ was prepared with a hydrothermal reaction method in an autoclave. The nano-belt morphology of MoO_3_ product would be in favor of the electrochemical accessibility of the redox active electrolyte to them. In the meanwhile, PANI was directly deposited onto Ti mesh current collector via a simple electrochemical polymerization method. The electrochemically deposited PANI was in a form of nano-fibers, which constitute a porous PANI nano-layer on the Ti micro-wires in the mesh. The effect of the presence of the redox active additive in the electrolyte on the electrochemical behaviors of both MoO_3_ and PANI electrode will be investigated, as well as the effect on the performance of the ASC that was assembled with them. This novel all-pseudocapacitive-asymmetric design with larger operating voltage is expected to generate a higher capacitive performance and it has a prospect to bridge the gap between dielectric capacitors and rechargeable batteries [20,21].

## 2. Experimental

### 2.1. Materials

Ferrous sulfate heptahydrate and ferric sulfate were purchased from Tianjin YongDa (Tianjin, China). Molybdenum powder was purchased from RHAWN (YiEn, Shanghai, China). The acetylene black and PTFE that were used for the electrode fabrication were purchased from MACKLIN (MACKLIN, Shanghai, China) and Taiyuan Lizhiyuan (LiYuan, Taiyuan, China), respectively. A porous film (PP/PE, EVOH, and Nylon membrane, Mitsubishi, Tokyo, Japan) was used as the separator in the capacitor. All of the reagents were of analytical grade and used without further purification.

### 2.2. Synthesis of PANI/Ti Electrode

The electrodeposition process was conducted with a three-electrode configuration. An electrolyte contains 0.5 M HCL and 0.2 M aniline was prepared and used as the electroplating solution. A saturated calomel electrode (SCE, INESA, Shanghai, China) and platinum foil (25 mm × 40 mm × 0.15 mm, Gaoss, Wuhan, China) were used as reference and counter electrode, respectively. Ti mesh (Kangwei, Hengshui, China) was used as a work electrode for the electroplating of conductive polymer, and the area being immersed into the electrolyte was 1 cm^2^. The cyclic voltammetry (CV) technique with a potential range of 0–0.9 V and a scan rate of 20 mV s^−1^ was adopted for the deposition of PANI. After seven cycles of CV, PANI (~0.5 mg) was deposited onto Ti mesh. A constant voltage of −0.2 V was applied on the electrode materials for 1 min in order to enhance the adhesion of PANI to Ti substrate. This process aimed to de-dope the PANI and made it shrink tightly to the Ti mesh substrate. The obtained PANI/Ti composite was then washed with distilled water and directly used as an electrode.

### 2.3. Synthesis of MoO_3_ Electrode

The MoO_3_ nanobelts were synthesized according to the method that was reported elsewhere [11]. Typically, 1 g of molybdenum powder was added into 10 mL DI water and then stirred in an ice bath. 20 mL H_2_O_2_ was added into the mixture drop by drop. When the liquid mixture became saffron yellow, it was transferred into a 50 mL Teflon-lined stainless steel autoclave and kept at 220 °C for 14 h. The obtained precipitation was filtrated, washed, and finally dried in a vacuum oven under 80 °C for 4 h. The electrode was fabricated by mixing the as-produced MoO_3_, acetylene black and PTFE with a weight ratio of 8:1:1 in ethanol, and the resulting slurry was spread onto a piece of Ti mesh with the same size as that for the electrodeposition of PANI.

### 2.4. Electrochemical Measurements 

The cyclic voltammetry (CV) and galvanostatic charge/discharge (GCD) tests for both PANI/Ti electrode and MoO_3_ electrode were performed with a typical three-electrode configuration. 0.1 M Fe^2+/3+^ redox ion pair was introduced into 0.5 M H_2_SO_4_ to form the redox active electrolyte. Bare 0.5 M H_2_SO_4_ was also prepared for reference. Asymmetric supercapacitor was assembled with both the transition metal oxide and conductive polymer electrodes. In order to balance of the quality of electricity of the two electrodes in ASCs, a mass ratio of 1:1.8 for MoO_3_ to PANI was adopted to assemble ASCs with 0.5 M H_2_SO_4_ electrolyte and 3:1 for ASCs with 0.1 M Fe^2+/3+^/0.5 M H_2_SO_4_ electrolyte, respectively. Electrochemical impedance spectroscopy (EIS) was obtained with a frequency range of 100 KHz to 10 m Hz and a 5 mV AC amplitude. All of the electrochemical tests were conducted on a CHI760e electrochemical workstation (CH Instruments, Shanghai, China). The specific capacitance (F g^−1^) (Cs is for the calculation from CV curves, while Cm is for GCD curves), energy density (E, Wh kg^−1^), and power density (P, kW kg^−1^) were calculated by the following equations:

(1) $$C_{S}=\frac{\int IdV}{2m\vartheta ∆V}$$

(2) $$C_{m}=\frac{I∆t}{m∆V}$$

(3) $$E=\frac{1}{2\times 3.6}C_{m}∆V^{2}$$

(4) $$P=\frac{3.6E}{∆t}$$

where *m* (g) is the mass of active material in the electrode in the three-electrode configuration (for ASC, total mass of the active materials in both electrodes), *I* (A) is the charge–discharge current, ϑ (V s^−1^) is the scan rate, ∆V (V) is the potential window (the IR (internal resistance) voltage drop is excluded), and ∆t (s) is the discharge time.

### 2.5. Characterization

The sample morphology was observed by scanning electron microscopy (SEM; Zeiss ULTRA plus, ZEISS, Jena, Germany). X-ray diffraction (XRD,) patterns were recorded on an X-ray diffractometer (UItima IV, Rigaku, TheWoodlands, TX, USA). The Raman spectra of the samples were obtained under a 633 nm laser light (Invia Renishaw Raman, Renishaw, Gloucestershire, UK). 

## 3. Results and Discussion

### 3.1. Morphology of PANI/Ti and MoO_3_

**Figure 1:**
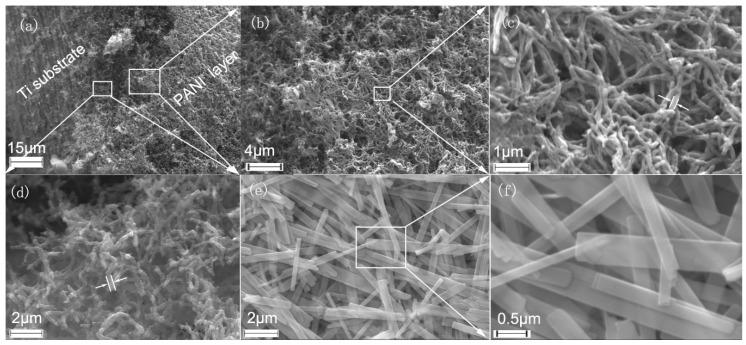
Scanning electron microscopy (SEM) images of polyaniline (PANI) nanofibers (**a**–**d**) and molybdenum oxide (MoO_3_) nanobelts (**e**,**f**).

The electrodeposited PANI nanofibers cover the whole Ti microwires in the mesh and form a porous layer, as shown in Figure 1a,b. The nanofiber observed on the outer surface of PANI porous layer has a diameter of about 100 nm (Figure 1c), which is half that of the PANI deposited just on the surface of Ti substrate presented in Figure 1d. The different diameter of the fibers possibly originates from the decreasing concentration of the monomer with the proceeding of the electro-deposition of PANI, where the fibers in outer surface of the PANI layer are polymerized and deposited at a lower concentration of monomers in the electrolyte near the electrode. Specifically, the porous layer structure of the electrochemical active PANI nanofibers could provide a relatively high specific surface area for the electrochemical interaction with the active species in the electrolyte, consequently promoting the capacitive performance of the PANI/Ti electrode. 

In comparison with the nanofiber morphology of the polymer electrode, the as-prepared MoO_3_ displayed a belt shape, as shown in Figure 1c,d. They have a width range of 30–700 nm and a length over 20 µm. Similar to the porous PANI nanofiber electrode, the MoO_3_ electrode materials also exhibit a porous structure and plenty of active sites on the edge of the nanobelts could afford them a highly active electrochemical interface with the electrolyte.

**Figure 2:**
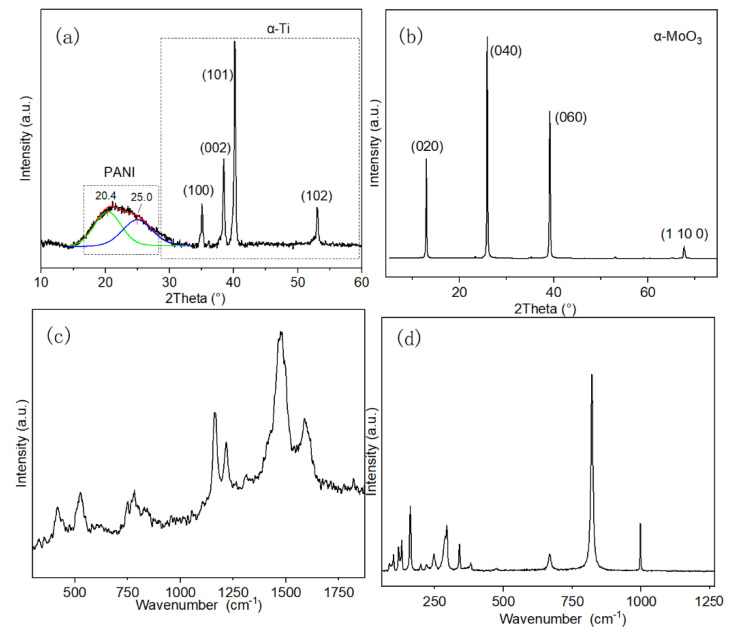
X-ray diffraction (XRD) spectra of PANI/Ti composite (**a**) and MoO_3_ (**b**); Raman spectra of PANI (**c**); and, MoO_3_ (**d**).

In the XRD pattern of PANI/Ti electrode, the broad diffraction peak at 15–30° is attributed to the parallel and perpendicular periodicity of the PANI polymer chain, as shown in Figure 2a. This peak could be deconvoluted into two peaks at 20.4° and 25.0°, which reveals the repetition of benzenoid and quinoid rings in PANI macromolecular chain, respectively [22]. Additionally, the presence of these two peaks could confirm the emeraldine salt form of the PANI product. The intense peaks recorded at 35.1°, 38.5°, 40.1°, and 53.1° represent (1 0 0), (0 0 2), (1 0 1), and (1 0 2) plane of the α-Ti substrate, respectively [23]. The XRD pattern of the MoO_3_ nanobelts reveals a high crystallinity of the product, which is in contrast with that of the conductive polymer, as shown in Figure 2b. The intense diffraction peaks at 13.0°, 25.9°, 39.2°, and 67.8° represent the (0 2 0), (0 4 0), (0 6 0), and (1 10 0) planes of orthorhombic crystal structure (α-MoO_3_), respectively. The peaks of (0 k 0) (where k = 2, 4 and 6) with so strong intensity reveal the anisotropic growth of the nanobelts [24]. Therefore, a strong orientation was preferred for the growth of MoO_3_ during the process of the hydrothermal reaction.

In the Raman spectra of PANI (Figure 2c), the bands that were detected at 400–1000 cm^−1^ originate from the deformation vibration of benzene rings [25]. The two bands at 1481 and 1588 cm^−1^ are relative to the C=N and C=C stretching vibration in the quinonoid units, respectively. The band at 1219 cm^−1^ is related to the benzene-ring deformation vibration, and the C–H in-plane bending vibration of semiquinonoid and benzenoid ring generates the band at 1163 cm^−1^ [26]. Figure 2d shows the Raman spectrum of MoO_3_ nanobelts. The intensive bands at 821 and 995 cm^−1^ are associated to the symmetric and asymmetric stretch of the terminal oxygen atoms, respectively. The band at 667 cm^−1^ is related to the asymmetric stretching of Mo–O–Mo bridge along the c axis [27]. The transition of the rigid chains generates the band at 161 cm^−1^, while the band at 293 cm^−1^originates from the wagging of the terminal oxygen atoms in the metal oxide.

### 3.2. Electrochemical Performance 

**Figure 3:**
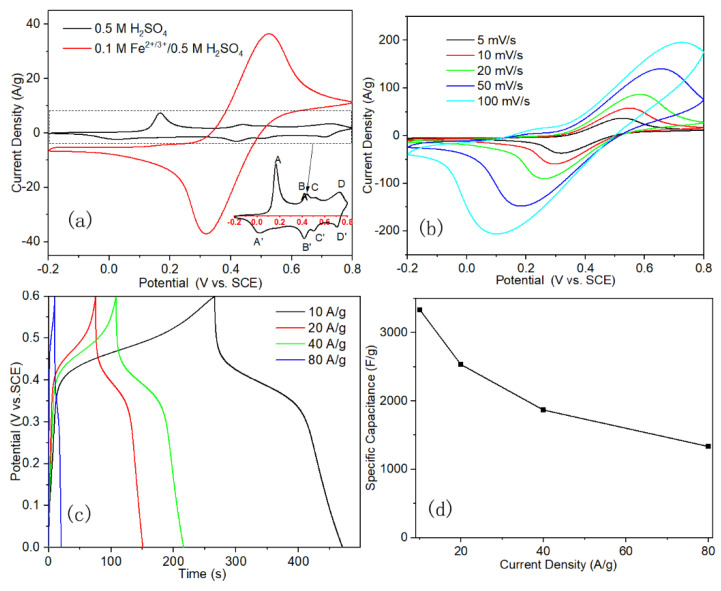
(**a**) Cyclic voltammetry (CV) curves of PANI/Ti electrode at 5 mV s^−1^ in different electrolyte, inset is the magnified CV curve in 0.5 M H_2_SO_4_; (**b**) CV, and (**c**) galvanostatic charge/discharge (GCD) curves of PANI/Ti electrode in 0.1 M Fe^2+/3+^/0.5 M H_2_SO_4_; and, (**d**) the dependence of specific capacitance of PANI/Ti electrode in 0.1 M Fe^2+/3+^/ 0.5 M H_2_SO_4_ on the current density.

The PANI/Ti electrode was directly used as a work electrode after the electro-deposition process. In comparison with those PANI synthesized with chemical oxidation methods and applied onto the current collector together with carbon black and adhesive, the electropolymerized PANI onto Ti mesh current collector could maintain the high electrochemical properties of PANI in the electrochemical devices. Besides, the porous structure of PANI layer that formed on Ti mesh has been optimized for the interaction between the electroactive materials and electrolyte during the electro-deposition process, and it could continue to take effect in the capacitors. Firstly, in order to study the effect of the presence of the Fe^2+/3+^ redox couple in the electrolyte on the performance of the electrode materials, PANI/Ti electrode was tested under three-electrode configuration in 0.5 M H_2_SO_4_ and 0.1 M Fe^2+/3+^/0.5 M H_2_SO_4_, respectively. CV curves that were recorded in different electrolyte were quite different from each other, as shown in Figure 3a. The area that was surrounded by the CV curve obtained in 0.5 M H_2_SO_4_ was much less than that in 0.1 M Fe^2+/3+^/0.5 M H_2_SO_4_, indicating a much larger capacitance of PANI in the redox active electrolyte. Moreover, the CV curve of the electrode changed a lot after the addition of Fe^2+/3+^ in the electrolyte. In neat sulfuric acid electrolyte, there are four pairs of redox peaks that are located at 0.17/0.03 V, 0.44/0.42 V, and 0.52/0.5 V and they are labeled as A/A’, B/B’, C/C’, and D/D’, respectively. A/A’ and D/D’ are related to the redox of leucoemeraldine (PANI reduced state, LE) to emeraldine (PANI half oxidized state, EB), and then from EB to prenigraniline (PANI totally oxidized state, PNB). Adjacent B/B’ and C/C’ are attributed to the formation of head-to-tail dimer [28,29,30]. In contrast, only one pair of peaks originated from the Fe^2+/3+^ redox couple could be observed in the CV curve that was obtained in the redox active electrolyte. These mean that the capacitance of the PANI electrode in the redox active electrolyte is mainly contributed from the electrochemical redox reaction between Fe^2+^ and Fe^3+^. The porous structure of the PANI electrode afforded the electrode with highly active interface for the electrochemical redox reaction of Fe^2+/3+^ ion pair, which can be described as the following reactions:

$P_{m}-xe+xA^{-}⇌P_{m}{}^{x+}A_{x}{}^{-}$; $Fe^{2+}-e^{-}⇌Fe^{3+}$  (a) (oxidation reaction)
$P_{m}+ye+xM^{+}⇌P_{m}{}^{y-}M_{y}{}^{+}$; $Fe^{3+}+e^{-}⇌Fe^{2+}$   (b) (reduction reaction)

PANI with conjugated double bonds is labeled as P_m_ (m is the polymerization degree), and A^−^ and M^+^ refer to anions and cations, respectively. The above oxidative p-doping reaction takes place in conducting PANI with both cations and anions being involved in the electrochemical redox processes of PANI [31]. The synergistic effect between PANI nanofibers and redox Fe^2+/3+^ ion pair promotes the capacitance and cycling stability of the electrode, which has also been demonstrated recently [17,18]. To further study the electrochemical performance of PANI/Ti electrode in the redox active electrolyte, more CV and GCD tests were conducted. The redox peaks shift from 0.32 /0.53 V at 5 mV s^−1^ to 0.1 /0.73 V at 100 mV s^−1^, which could be due to the slower ion transmission on the interface of PANI nanofiber and electrolyte in comparison with the electron transfer inside the bulk PANI structure at larger scan rate, as shown in Figure 3b [10].

Specific capacitances that were calculated from CV curves in Figure 3b by Equation (1) are listed in Table S1 in the Supplementary Materials. The GCD curves at different current densities were also shown as Figure 3c. The corresponding specific capacitances calculated by Equation (2) were 3330, 2530, 1867 and 1330 F g^−1^ at 10, 20, 40, and 80 A g^−1^, respectively, as shown in Figure 3d. The specific capacitance in the redox active electrolyte was 14 times higher than that in neat sulfuric acid electrolyte, which is only 230 F g^−1^ at 10 A g^−1^. 

**Figure 4:**
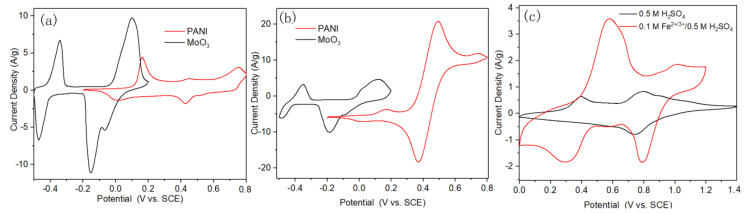
CV curves of PANI and MoO_3_ electrode materials at a scan rate of 2 mV s^−1^ in (**a**) 0.5 M H_2_SO_4_ and (**b**) 0.1 M Fe^2+/3+^/ 0.5 M H_2_SO_4_, respectively; and, (**c**) CV curves of asymmetric super-capacitors (ASCs) assembled with both PANI and MoO_3_ electrodes at 5 mV s^−1^ in different electrolytes.

ASCs were assembled with PANI/Ti as positive electrode and MoO_3_ as negative electrode. Two ASCs were assembled with electrolyte of 0.5 M H_2_SO_4_ and 0.1 M Fe^2+/3+^/0.5 M H_2_SO_4_, respectively, in order to explore the effect of the presence of the Fe^2+/3+^ redox active additive in the electrolyte on the performance of the capacitors. Two pairs of redox peaks appear in the CV curve of MoO_3_ in 0.5 M H_2_SO_4_, which contribute a lot of pseudo-capacitance to the electrode and the corresponding specific capacitance calculated by Equation (1) was 1243 F g^−1^, as shown in Figure 4a. The obtained capacitance of single MoO_3_ electrode is higher than most reported values in literatures, as shown in Table 1. The oxidation peak at 0.1 V and its counterpart reduction peak with a shoulder at −0.15/0.07 V are related to the MoO^6+^/MoO^5+^ redox reaction. Another pair of redox peaks at −0.34/0.47 V could be possibly due to the further redox reaction of Mo centers [32]. It can be also found that the current density of the redox peaks of PANI in 0.5 M H_2_SO_4_ was much weaker than that of MoO_3_. After adding Fe^2+/3+^ active additive into the electrolyte, the capacitive performance of PANI was significantly improved, as shown in Figure 4b. In the meanwhile, the response current density of MoO_3_ changed little in comparison with that in 0.5 M H_2_SO_4_, which indicated the different effect of the same redox additive in the electrolyte on the different electrodes. This is caused by the electrochemical redox reaction of the Fe^2+/3+^ redox couple mainly occurring in the potential window of PANI, as demonstrated in Figure 3a. ASCs of PANI//MoO_3_ were assembled with electrolyte of 0.5 M H_2_SO_4_ and 0.1 M Fe^2+/3+^ /0.5 M H_2_SO_4_, respectively. Precisely, Ti mesh (80 mesh) was used as the current collector, PANI and MoO_3_ electrodes were separated by a membrane with a thickness of 150 μm, and then all of the electrodes and membrane were immersed with the electrolyte. The work electrode was connected to the Ti mesh that was deposited with PANI, and the reference and counter electrode were connected to the MoO_3_ electrode. Figure 4c displays the CV curves of ASCs with different electrolytes at the same scan rate of 5 mV s^−1^, where the current density was significantly promoted with the presence of 0.1 M Fe^2+/3+^ in the electrolyte. Precisely, the specific capacitance that was obtained from GCD tests was 63 F g^−1^ at 1 A g^−1^ in bare sulfuric acid electrolyte, which is much less than that measured at the same current density in 0.1 M Fe^2+/3+^ modified electrolyte (197 F g^−1^).

**Figure 5:**
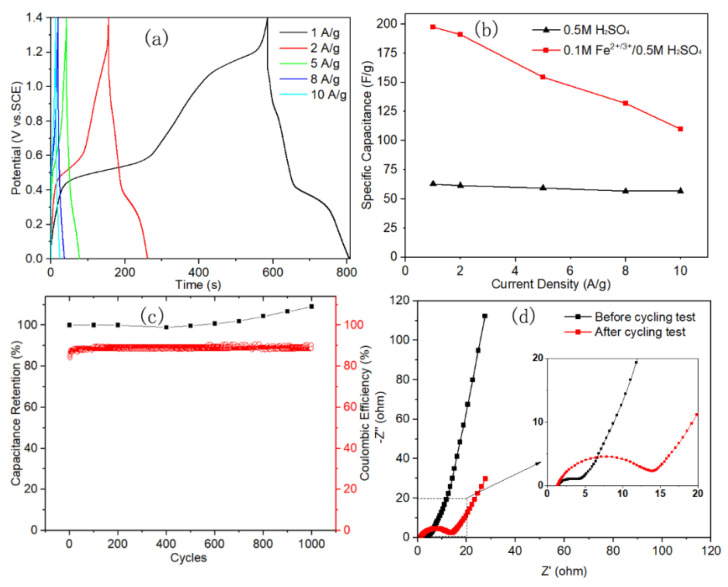
(**a**) GCD curves of ASC at different current densities; (**b**) specific capacitance of ASC in different electrolytes; (**c**) capacitance retention and the columbic efficiency of 1000 cyclic test; and, (**d**) electrochemical impedance spectroscopy (EIS) spectra before and after cycling test.

The electrochemical performance of ASC with redox active electrolyte was comprehensively explored with GCD tests under various current density and EIS tests before and after the 1000 charge–discharge cyclic stability test. Two discharge platforms could be observed in the GCD curves recorded under 1 A g^−1^, as shown in Figure 5a. This coincides with the corresponding CV curve in Figure 4c, which exhibits two pair of redox peaks, despite that some peaks were too weak to be interpreted in the corresponding GCD curves. The energy density value is proportional to the square of the working potential window, according to Equation (3). With the appropriate design, the operation potential range of the ASCs was enlarged up to 1.4 V in the aqueous electrolyte; therefore, a high energy density of 54 Wh kg^−1^ at 0.9 kW kg^−1^ and 30 Wh kg^−1^ at 36 kW kg^−1^ power density were achieved. Figure 5b shows the specific capacitance calculated from GCD curves in Figure 5a. Notably, when the current density increased by 10 times to 10 A g^−1^, a specific capacitance of 110 F g^−1^ was achieved for the ASC with the redox active electrolyte, and it is still twice more than those that were measured in the bare sulfuric acid electrolyte. Specific capacitances that were calculated from CV tests are listed in Table S1 in the supporting information. Besides, the capacitive performance was compared with other results for the ASCs containing MoO_3_ or MoO_3−x_ electrode, as shown in Table 1, which indicated its relatively high energy density at the largest power density. The cyclic stability of the ASC was also studied. The capacitance of the ASC was even increased to 109% after 1000 cyclic tests, as shown in Figure 5c. This is in contrast with our recently reported result of PANI symmetric capacitors with the similar redox active electrolyte, which was so unstable that only ~50% capacitance was retained after 1000 cycles [18]. Moreover, the coulombic efficiency was increased from 84% for the first cycle and remained stable at a high level of nearly 90% after 100 cycles, indicating a good reversibility of the ASC [33].

The EIS test before and after cyclic test was carried out to explore the change of electrochemical process of the capacitor. Figure 5d shows the resulting Nyquist plots, where the intersection point of the curve at x-axis that represents the equivalent series resistance (Rs) of the capacitor was almost unchanged after the multi-cycling test, indicating a high stability of ASC in the 0.1 M Fe^2+/3+^/0.5 M H_2_SO_4_ redox electrolyte. The diameter value of the semicircle area that was related to charge transfer resistance (Rct) was increased approximately four times from 3.3 Ω to 12.5 Ω, which could be caused by the volume change of both the electrochemical active electrode materials as their chemical state various with the charge/discharge process during the multi-cycle test. The sustainably increasing of the specific capacitance of the ASC with the cycle number (Figure 5c) could also be due to the gradually increasing extent of the infiltration of both the porous PANI nanofiber and MoO_3_ nanobelt electrode by the redox active electrolyte during the multi-cycling test.

## 4. Conclusions

In this work, two typical kinds of electrochemical active electrode materials, i.e., transition metal oxide (MoO_3_) nanobelts and conductive polymer (PANI) nanofibers, were fabricated with either the hydrothermal reaction method or electro-synthesizing approach and utilized to assemble ASCs. The promoted capacitive performance of both the electrode materials in the redox active electrolyte containing Fe^2+/3+^ additives was demonstrated in comparison with their electrochemical behavior in the common sulfuric acid electrolyte. By adding only a few redox active additive (0.1 M Fe^2+/3+^) in the electrolyte, the specific capacitance of PANI nanofiber electrode was promoted by 14 times, in contrast with little change of the electrochemical behavior of MoO_3_ nanobelt electrode in the same case. The ASC assembled with PANI nanofibers as positive electrode and MoO_3_ nanobelts as negative electrode in 0.1 M Fe^2+/3+^/0.5 M H_2_SO_4_ redox active electrolyte exhibits a high energy density of 54 Wh kg^−1^ at a power density of 0.9 kW kg^−1^ and specific capacitance of 197 F g^−1^, which is three times more than that measured in normal 0.5 M H_2_SO_4_. Additionally, the multi-cycle test demonstrates its high cyclic stability and 109% of the pristine capacitance remained after completing 1000 charge–discharge cycling tests. The PANI//MoO_3_ ASC with such reliable capacitive performance indicates its promising application as a kind of energy storage device.

## Figures and Tables

**Table 1:** Performance of MoO_3_ single electrode and ASCs assembled with MoO_3_ electrode.

Sample	Electrolyte	Capacitance/F g^−1^ (Current Density)	Energy Density (Wh/kg)	Power Density (W/kg)	Testing Configuration	Ref.
MoO_3_	H_2_SO_4_	1243 (2 mV/s)	-	-	half-cell	present
MoO_3_	Li_2_SO_4_	280 (1 mV/s)	-	-	half-cell	[34]
MoO_3−x_	H_2_SO_4_/EG	1230 (5 A/g)	-	-	half-cell	[10]
MoO_3_	H_2_SO_4_	560 (1 A/g)	-	-	half-cell	[35]
AC//MoO_3−x_	H_2_SO_4_/EG	313 (1 A/g)	111	803	full-cell	[10]
AC//MoO_3_	Li_2_SO_4_	30 (2 A/g)	45	450	full-cell	[34]
CNTs/MnO_2_//MoO_3_/PPy	Na_2_SO_4_	~47 (0.24 A/g)	21	220	full-cell	[11]
PANI//MoO_3_/PANI	H_2_SO_4_	49 (5 mV/s)	10	53	full-cell	[36]
PANI//MoO_3_	H_2_SO_4_	518 (0.5 A/g)	72	254	full-cell	[35]
GrMnO_2_//GrMoO_3_	Na_2_SO_4_	~90 (1 A/g)	43	276	full-cell	[37]
PANI//MoO_3_	H_2_SO_4_/Fe^2+/^^3+^	197 (1 A/g)	54	900	full-cell	present

## Supplementary Materials

The following are available online at https://www.mdpi.com/2073-4360/12/10/2303/s1, Table S1: Specific capacitance of PANI//MoO_3_ ASCs and PANI/Ti single electrode in 0.1 M Fe^2+/3+^ /0.5 M H_2_SO_4_ calculated from GCD tests (Cm) and CV curves (Cs).
