# Supplementary material for: Electrodeposited Polyaniline Nanofibers and MoO3 Nanobelts for High-Performance Asymmetric Supercapacitor with Redox Active Electrolyte
Source: Polymers (Basel). 2020 Oct 8;12(10):2303. doi: 10.3390/polym12102303 (PMC7600686; doi:10.3390/polym12102303)
Supplement: Supplementary file 1 [file polymers-12-02303-s001.pdf]

Support information

Electrodeposited Polyaniline Nanofibers and MoO_3 Nanobelts for High-Performance Asymmetric Supercapacitor with Redox Active Electrolyte

Wei Meng [1], Yanlin Xia [1], Chuanguo Ma [2] and Xusheng Du [1,*]

[1] Institute of Advanced Wear & Corrosion Resistance and Functional Materials, Jinan University, Guangzhou, 510632, China

[2] Guangxi Key Laboratory of Information Materials, Guilin University of Electronic Technology, Guilin, 541004, China

* Correspondence: xdusydjn@email.jnu.edu.cn; Tel.: +86-20-8522-2151

Table S1. Specific capacitance of PANI//MoO_3 ASCs and PANI/Ti single electrode in 0.1 M $Fe^{2+/3+}$ /0.5 M H_2SO_4 calculated from GCD tests (Cm) and CV curves (Cs).

	Scan Rate (mV/s)	Cs (F/g)	Current Density (A/g)	Cm (F/g)
PANI//MoO_3	5	102	1	197
	10	104	2	194
	20	97	5	179
	50	65	8	188
	100	38	10	189
PANI	5	1400	10	3330
	10	1295	20	2530
	20	1080	40	1867
	50	803	80	1330
	100	565	-	-
